# Recycling of Epoxy/Fiberglass Composite Using Pyridine

**DOI:** 10.3390/polym17111513

**Published:** 2025-05-29

**Authors:** Alexander E. Protsenko, Alexandra N. Protsenko, Olga G. Shakirova, Victor V. Petrov

**Affiliations:** Department of Chemistry and Chemical Technology, Komsomolsk-na-Amure State University, Komsomolsk-on-Amur 681013, Russia; protsenko.chem@gmail.com (A.N.P.); shakirova_olga@mail.ru (O.G.S.); petrovpng@mail.ru (V.V.P.)

**Keywords:** polymer composite material, fiberglass reinforced plastic, epoxy matrix, solvolysis, recycling, subcritical fluid, pyridine, fiberglass, strength

## Abstract

This study presents a new approach to chemical processing using pyridine-based solvolysis to produce high-quality glass fiber from epoxy composites. Pyridine was chosen due to its solubility parameter, which precisely matches the parameters calculated for the epoxy matrix segment. Experiments with exposure in a pyridine medium demonstrated effective swelling and the potential for destruction. The solvolysis experiments were conducted in a round-bottomed flask with a reflux condenser and stirrer, under ambient conditions (20 °C) until the boiling point was reached (115.2 °C). Additionally, data from experimental studies conducted at subcritical temperatures before reaching 280 °C are presented. The dependences of changes in the mass of composites on time and temperature during the solvolysis process were determined. The tensile strength of the recovered fibers was examined, and thermogravimetric analysis was used to determine their properties. Fiberglass recovered at the boiling point is characterized by 91% tensile strength and 20% residual degradation products on the surface. The residual strength of fiberglass-reinforced plastic (FGRP) is 70.3%. The use of subcritical pyridine helps improve the quality of plastic products made from recycled fibers. This process retains 93% of the residual tensile strength for fibers that have been processed at 250 °C for two hours. Recycled fibers also contain 2.82% organic components on their surfaces. Using this material results in an increase in flexural strength of FGRP by 16.1%, compared to the reference samples.

## 1. Introduction

Polymer composite materials (PCMs) are essential in today’s world due to their unique properties [1]. Composites are widely used in various fields of human life and industry [2,3,4,5,6,7]. PCMs based on thermosetting matrices, such as epoxy, polyester, and vinyl ester, are the most commonly used [8,9,10]. These matrices are characterized by their ability to form stable three-dimensional macromolecular networks as a result of curing. The formation of such a structure also leads to difficulties in utilizing and recycling these materials. At the same time, the use of these composites is increasing every year. The life cycle of these materials can reach 50 years, so the issue of recycling is not a major concern at the moment [3,11]. However, the amount of reactive plastic waste is expected to significantly increase in the near future [12]. Studies [13,14,15] presented results from reusing end-of-life products in another application. However, this approach does not solve the problem of composite waste accumulation. Instead, it simply allows society to use composite materials over a longer period. That is why research and development of thermosetting composite recycling is an acute issue corresponding to priority areas of scientific and technological development in different countries around the globe [2].

To date, there are three main methods for recycling end-of-life thermoset PCM products [14,16,17,18,19,20,21]: mechanical, physical and chemical. Due to the simplicity of hardware design mechanical [16,17,22] and physical methods [18,23,24,25,26] have become the most widely used ones. However, the disadvantage of the mechanical approach is that it is impossible to recycle fillers in their original form. As a result, fillers lose strength and technological properties during the grinding. Additionally, it is not possible to remove the cross-linked polymer from the surface of the filler due to the grinding. This means that this method does not address the issue of man-made waste accumulation. At the same time, there are approaches where end-of-life composite products to optimize the recycling process. For example, in fluidized bed processes [27]. High-temperature pyrolysis of polymer composites has become the most popular ones among physical recycling methods [28,29]. However, this method has some disadvantages, including the production of large amounts of hazardous volatile products and weakening of filler strength. Despite these drawbacks, it is a versatile method that allows the recycling of various polymer matrices.

The chemical method is the most promising one compared to the other methods [6,14,18,30,31,32]. As a result of the solvent treatment of the material under special conditions, it is possible to obtain the reinforcing filler. Additionally, a solvolysis liquid is produced as a result of the process. The composition of this liquid includes different products from the chemical destruction of polymers, which could be of use in various industries. There are also some known works where a polymer matrix has been created from products contained in the solvolysis liquid [33,34]. Liu et al. presented a way to use the technique of controlling the degree of crosslinking to recycle imine bond of the crosslinked elastomeric vitrimers [35] and to achieve self-healing effects [36].

Various compounds are used as solvents in solvolysis processes. Modern equipment for these processes makes it possible to work with solvents in different states: liquid, steam, subcritical and supercritical fluids [6,37,38]. The most commonly used and cheapest solvent is water [31,39,40], but under normal conditions, it is not very effective for thermosetting polymers. However, water in its supercritical state is one of the most efficient solvents. The main disadvantage of this state of water is its high critical temperature, which leads to high energy costs and requires the use of special equipment. Therefore, solvents with lower critical temperatures and pressures are more acute. Alcohols, for example, are widely used in the research of recycling processes of polymer matrices [41,42,43]. Ethanol effectively allows the epoxy matrix to be removed at 250 °C over a 10 h’s period [44,45]. When the temperature rises up to 280 °C, the process time is reduced to 4 h. In addition, epoxy matrices can be destroyed at the above mentioned temperature, as well as epoxy-vinyl ester matrices, which do not degrade at lower temperatures. Other organic solvents, such as methanol, acetone, glycerin, and ethylene glycol are also used. However, there have been few studies on the use of nitrogen-containing compounds for this purpose.

As a part of the research, it was found that nitrogen-containing compounds can significantly reduce the time and temperature required for solvolysis [45,46,47]. Catalytic effect of a complex compound consisting of copper(II) chloride and 2,3,5-triphenyltetrazolium chloride was studied [45]. However, the main disadvantage of the approach is the instability of the compound in the reaction medium. Additionally, the study investigated the effect of organic cations on the solvolysis process of epoxy matrices.

In [46,48] the methods allowing to obtain the recovered fillers through solvolysis in an amino alcohol environment are presented. However, the main disadvantage of the latter methods is that they need alkaline catalysts for reaction process.

Therefore, it is interesting to study the effect of different nitrogen-containing organic solvents on the decomposition of thermosetting PCM in order to obtain recovered reinforcing fillers.

## 2. Materials and Methods

### 2.1. Materials

The object of this investigation was a fiberglass-reinforced plastic (FGRP) composed of cross-woven fiberglass fabric 1250-T30-290 (Umatex, Moscow, Russia) and a two-component epoxy resin system SR8100/SD8824 (Sicomin, Châteauneuf-les-Martigues, France) based on the bisphenol-A and -F and a multifunctional amine hardener. FGRP and its analogs finds widespread application in diverse fields [3,49,50,51,52].

It should be said that 99% pure solvents were used for the study of solvolysis and swelling: aniline, benzene, xylene, toluene, pyridine, i-butanol, butyric acid, n-butanol methanol, ethanol, acetic acid, phenol.

### 2.2. Methods

The nine-layered composite panels were fabricated via the vacuum-assisted resin transfer molding (VaRTM) technique. The SVI-20-43 system (MSH Techno, Moscow, Russia) was employed to reach the goal. Afterwards the FGRP specimens underwent post-curing at 120 °C for a duration of 2 h to achieve optimal material properties.

The same techniques and the binder were used to produce FGRP with the recovered fillers. All the fabrics had the same size and shape as the original samples. The recovered fiberglass fabric samples were then dried in a convection oven at 120 °C for two hours to remove any volatile products. After that, they were used in the VaRTM process.

The swelling of the samples was investigated according to ISO 175:2010 [53]. Samples with a size of 20 × 20 × 3 mm were kept at a constant temperature of 25 °C for a specific time in the solvents under study in a thermostat. Before starting the experiment, each sample was weighed. After the predetermined time, the samples were removed from the reaction vessel and placed on the filter paper. They were then weighed again to determine the difference in mass compared to the initial weight.

Solvolysis at the normal pressure was performed in a round-bottom flask with a reflux condenser and continual stirring. Each experiment included a composite sample weighing 10 g (5 × 5 cm). The treatment was conducted over a range of temperatures from room temperature to the boiling point of the solvent.

Subcritical solvolysis was carried out in a laboratory reactor made of fluoroplast with a volume of 25 mL. A 2 g composite sample and 10 g of pyridine were added to the reactor. Then, the fluoroplastic insert with a tightly sealed lid was placed into a metal flask. The solvolysis was performed for various times in the temperature range up to 280 °C. A reactor with a volume of 250 mL was used to process large samples weighing 100 g each. of the recovered fiberglass were used to obtain FGRP.

In all solvolysis experiments, mass changes were calculated in the same way as in the swelling method. Error bars represent the standard deviation of at least five replicates.

Thermal analysis of the samples was performed using an STA 409 PC Luxx simultaneous thermal analyzer manufactured by NETZSCH-Gerätebau GmbH (Selb, Germany). Thermogravimetric (TG) and differential scanning calorimetric (DSC) data were recorded during the experiment. Analysis was carried out using corundum ceramic crucibles, and heating was performed at a rate of 10 K/min in air.

The microstructural analysis was conducted using a S-3400N (Hitachi Ltd., Tokyo, Japan) scanning electron microscope (SEM) with a tungsten cathode electron gun. The secondary electron detector with the accelerating voltage of 5 kV and backscattered electrons with the 30 kV were used for the measurements.

The IR absorption spectra of the compounds were acquired using an IRAffinity-1S FTIR spectrometer (Shimadzu, Kyoto, Japan), in the range of 400–4000 cm^−1^ with 1 cm^−1^ resolution and a 30,000:1 signal-to-noise ratio (peak-to-peak).

The quantitative and qualitative composition of the polymer matrix degradation products was analyzed with the help of a Shimadzu GCMS-QP2010 Ultra chromatography-mass spectrometry system. An RTX-5MS column with a length of 30 cm was used for the analysis. The oven temperature was heated from 30 °C to 300 °C at a rate of 5 °C per minute, followed by a holding time of 25 min. The injector temperature was set at 280 °C. A volume of 2 µL was injected using an AOC-20i autosampler, and no splitter was used. Helium 6.0 was used as carrier gas.

The microhardness was assessed using a Vickers method (Shimadzu HMV-2) with a force of 98.07 mN and a duration of 10 s. To ensure accuracy, ten measurements were taken in each area of the composite sample.

Tensile strength of elementary glass fibers was carried out in accordance with ISO 11566:1996 [54]. Elementary fibers were isolated from the bundle of thread. The fibers were glued into a frame with a hole size of 25 × 10 mm. The optical microscope Eclipse MA200 (Nikon, Shinagawa City, Japan) was used to determine the diameter of the fiber. The breaking load was determined using the ZQ-21A (Zhiqu, Haikou, China), test bench with a DS2-2N (Zhiqu) dynamometer.

The flexural strength of FGRP samples was tested for three-point bending in accordance with ASTM D790-10 [55] using Instron 3382. The size of the sample was 50 × 15 × 2 mm, and 2 mm radius supports were used for the tests. These supports were installed at a distance of 32 mm, and the loading speed was 1 mm/min.

## 3. Results and Discussion

To select an effective solvent for the recycling process of polymer composites with thermoset matrices, various nitrogen-containing solvents were investigated. The selection was based on calculations of the Hildebrand solubility parameter using the following equation [56,57]:

$$\delta^{2}=\frac{\sum_{i}{∆E}_{i}^{*}}{N_{A}\sum_{i}{∆V}_{i}},$$

where ΔΕ* is the cohesive energy of the liquid or the repeating unit of the polymer [58], *N_A_* is Avogadro’s number, Δ*V_i_* is the van der Waals volume of the *i-*th atom included in the repeating unit of the polymer [58].

**Figure 1:**
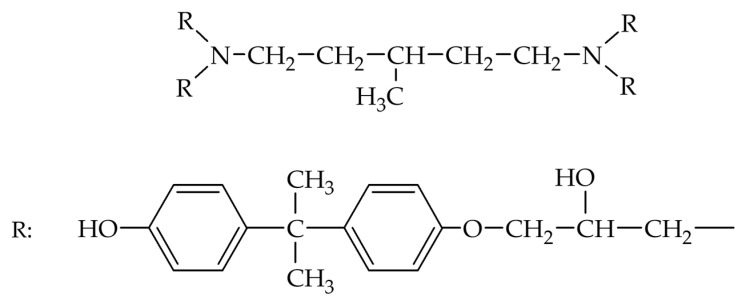
The structural formula of the epoxy matrix was used to calculate the solubility parameter.

The calculation of this parameter was performed for a repeating unit of the epoxy polymer chemical structure (Figure 1).

The calculation results are presented in Table 1, which also includes a list of solvents commonly used in the chemical industry [58]. The criterion for selecting a suitable solvent is the proximity of its solubility parameter to that of the polymer, with −2 < Δ*δ* < 2 considered satisfactory.

**Figure 2:**
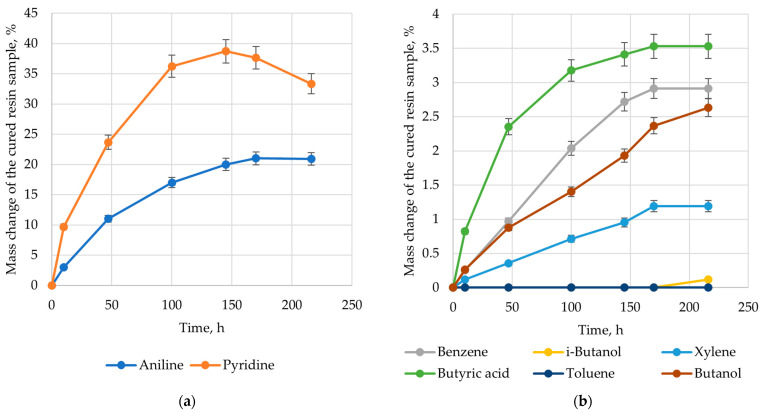
Mass change in the cured resin sample due to the exposure in the solvent: (**a**) aniline and pyridine; (**b**) benzene, i-butanol, butanol, xylene, toluene, and butyric acid.

A plot of mass change for epoxy plastic as a function of exposure time was obtained based on the study of swelling processes in selected solvents under normal conditions (Figure 2).

According to the data presented in Figure 2, the epoxy resin demonstrates varying degrees of swelling in different solvents. The nitrogen-containing solvents, especially pyridine and aniline, are of great interest. A swelling degree of 21% was recorded after 170 h in an aniline for the epoxy polymer. Upon further exposure to aniline, the mass of the material sample remained unchanged. Pyridine was of greatest interest, with a maximum swelling of 38% recorded after 140 h. As the exposure time increased, the mass of the sample decreased, most likely due to the destruction caused by swelling. The other solvents are of limited interest due to the low swelling degree of the epoxy polymer. The experimental data also confirmed the polymer solubility theory for thermoset epoxy. The most promising solvent is pyridine, with a Δ*δ* value of 0.005 (cal/cm^3^)^0.5^. The experimental data obtained for the swelling process are consistent with the results of previous studies, where amino alcohols were used as solvents [46,47]. Previous studies [45] have demonstrated the potential of using copper(II) complexes with nitrogen-containing organic ions as catalysts for recycling epoxy matrix composites in ethanol.

At the next stage, degradation of 10 g composite samples was investigated at elevated temperatures and ambient pressure in a round-bottom flask with a reflux condenser and continuous stirring. The maximum temperature used in this experiment was the boiling point of the solvent. Although effective swelling of the epoxy matrix was observed in aniline, its use at elevated temperatures could result in undesirable side reactions. This fact is caused by the high reactivity of the aniline’s amino group, which may result in the formation of aniline derivatives from epoxy matrix degradation products and aniline polymerization [59,60]. Thus, further studies of the solvolysis of polymer composites with an epoxy matrix were carried out in a pyridine medium.

**Figure 3:**
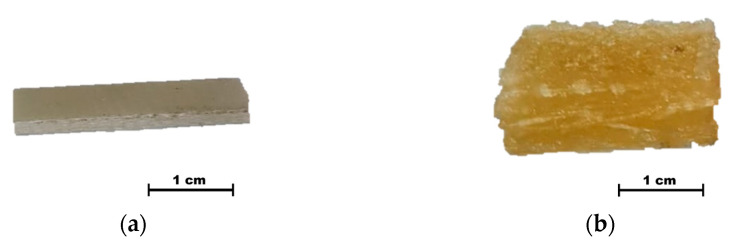
Samples before (**a**) and after pyridine treatment (**b**).

The use of pyridine as a solvent, with Its boiling point at 115.2 °C, reduced the duration of the solvolysis process to 1 h. Upon heating, there was a significant intensification of the swelling of the polymer matrix. The temperature in the flask reached 105.0 °C after five minutes of heating the sample. The thickness of the sample increased fourfold (Figure 3). During the solvolysis process, the color of the composite material changed from a pale green to a yellow-brown shade. A suspension of particles was also formed in the solution.

**Figure 4:**
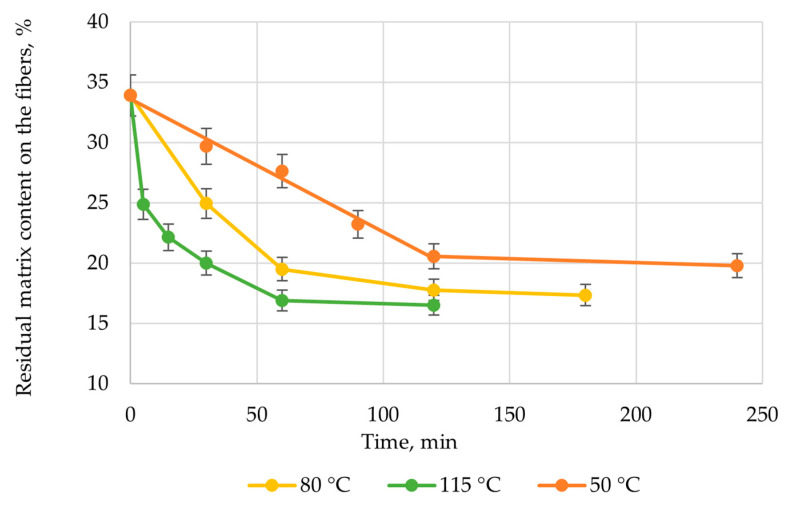
Dependence of the residual content of the epoxy matrix on temperature and time of solvolysis.

The effect of the solvolysis temperature in a pyridine medium on the rate of polymer matrix destruction has also been investigated. The process was carried out at temperatures of 50, 80, and 115.2 °C. A summary analysis of the experimental data allowed us to create Figure 4.

As a result of the destruction of the polymer matrix, separate fabric sheets of reinforcing filler were obtained. The residual matrix content in the fabric after the solvolysis process was recalculated using equation:

$$R_{r}=\frac{F_{0}\cdot R_{E}}{F_{E}},$$

where $R_{r}$ is the recalculated residual matrix content on the fibers, wt.%; $F_{0}$ is the initial filler content determined using TG analysis, wt.%; $F_{E}$ is the filler content of the treated composite material determined using TG analysis, wt.%; $R_{E}$ is the residual matrix content on the fibers of the treated composite material determined using TG analysis, wt.%.

The residual content of organic products on the fibers after the solvolysis process, according to TG analysis, was 20 wt.%.

The degradation process proceeded most intensively during the first 30 min at the boiling point. Thereafter, a plateau was reached, with no further increase observed after 1 h. Extended exposure of polymer composite samples in the reaction medium did not enhance polymer degradation.

The destruction process occurring at the boiling point of pyridine was almost 18 times faster in the initial stage compared to solvolysis at a temperature of 50 °C. This confirmed the need to increase the temperature to intensify the process. Furthermore, the depth of processing improved with increasing temperature. The residual content of the epoxy matrix in the recycled fibers decreased from 23.0 wt.% to 20.4 wt. The delamination of the samples occurred earlier when the process was carried out at the boiling point. After 120 min of treatment at 50 °C, complete delamination was recorded. At the boiling point, the sample delaminated after 30 min. Despite the composite samples were delaminated and individual fiberglass fabrics were obtained, increasing the duration of the process did not lead to complete purification of the fibers. However, these recovered fillers can already be used to produce composites.

**Figure 5:**
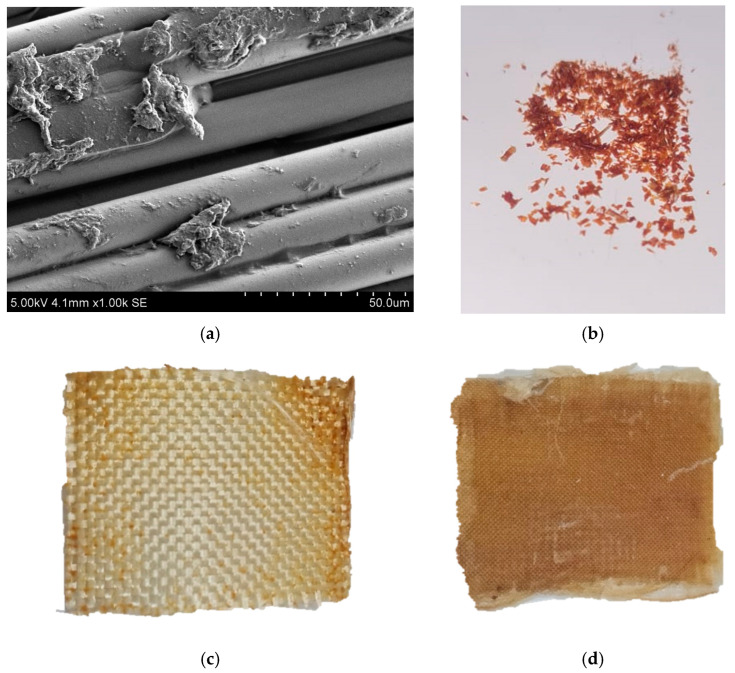
SEM of the fiberglass recovered in pyridine (**a**) and photo of residue of non-destructed polymer (**b**), recovered fiberglass (**c**) after solvolysis at the boiling point after 1 h; FGRP with recovered fiberlass (**d**).

The remnants of the polymer matrix on the recovered fillers were identified by SEM analysis (Figure 5a). The particle size on the fiber surface ranged from 0.75 to 5 µm. Additionally, extensive polymer regions between some fibers were observed, which provide greater rigidity to the recycled fabric compared to the original material. The polymer layers between fibers exhibited a smooth, streamlined morphology, similar to a solidified melt. This indicates that matrix destruction was caused by swelling and dissolution of the components. This pattern differs significantly from the destruction of laminates by adhesive bonding, where the rivulet structure and the torsion bars on the surface of the epoxy thermosetting material are dominant [61]. In some areas, large and irregularly shaped matrix fragments were observed, with sizes that can reach up to 50 µm in diameter. Furthermore, the presence of free fibers indicates that they may be impregnated with the binder to create a composite material based on recycled fiberglass sheets.

Also, a powdery sediment formed at the bottom of the flask (Figure 5b) at the end of the solvolysis process. FTIR study established the identity of the initial matrix of composite material (Figure S1), residues on the surface of fibers (Figure S2) and sediment from the flask (Figure S3). The main peaks of valence vibrations corresponding to the epoxy matrix were identified in the spectrum: a broad band of intermolecular H-bonds in dimers and polymers ν(OH) at around 3400 cm^−1^ and δ(OH) between 1300 cm^−1^ and 1240 cm^−1^. Additionally, peaks corresponding to fluctuations in the benzene ring were observed between 1600 cm^−1^ and 1450 cm^−1^. Due to the incorporation of pyridine into the matrix structure, its characteristic peaks were observed in the region of 3087–3028 cm^−1^ ν(CH), 1665–1590 cm^−1^ δ(CH) and 1070–1040 cm^−1^ δ(CH) on the spectrum.

The study of the solvolysis liquid by GCMS showed that there were no low-molecular-weight destruction products from polymer matrices in the composition, except for acetone and ethanol. More than 80% of the liquid was attributed to pyridine in terms of the area of all peaks. Considering that acetone was in the solution, it can be assumed that it forms as a result of the rupture of C-N and C=O bonds in macromolecular chain fragments. Under the same conditions, according to available data, no C-C bond breaks occur. The solution turned yellow, which may indicate the presence of oligomeric components. Therefore, it can be assumed that swelling of the samples was most likely limited. Pyridine, a polar aprotic solvent, penetrated the epoxy matrix structure, causing swelling. The solvent boiling induced thermal stresses in the swollen matrix. Local temperature and vapor pressure fluctuations caused microcracks and physical structure destruction. This led to disintegration of large pieces into small particles preserving the chemical structure of original polymer. The obtained data suggested that the mechanism of polymer matrix destruction in pyridine caused by the mechanical and chemical destruction of cross-linked macromolecular structures due to their swelling. These results are in good agreement with those from the study on the recycling of printed circuit boards based on brominated epoxy resins, which found that swelling in organic solvent environments can lead to destruction [62].

The tensile strength of elementary fibers from fiberglass samples obtained under solvolysis conditions in a pyridine medium at atmospheric pressure was studied (Table 2). The recovered fibers had a strength of 91% of the virgin fibers.

**Figure 6:**
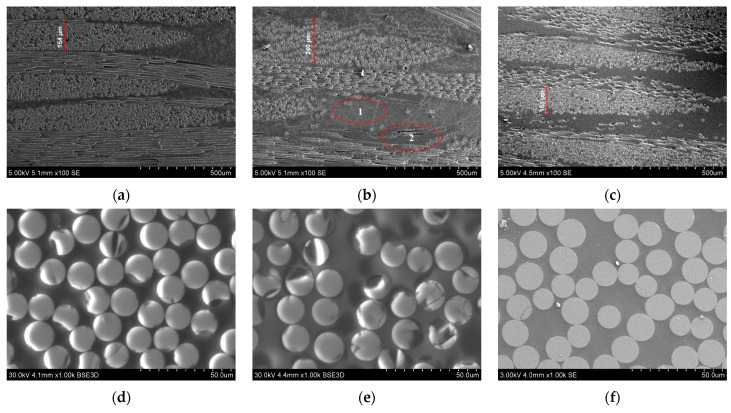
SEM of FGRP based on: (**a**,**d**) virgin fiberglass; (**b**,**e**) recovered in pyridine under normal pressure and temperature of boiling point and (**c**,**f**) 250 °C in autoclave. Bubles at the Figure (**b**) correspond to different Phases.

The recovered fabric fillers were used to produce polymer composite materials based on an epoxy binder using the VaRTM method. SEM analysis of the composite samples made with recovered fillers was performed (Figure 6). It was found that due to the fact that in case (b), the matrix was not completely removed and the sample swelled, the fiberglass yarns significantly increased in size. Residual products prevent compaction of elementary fibers inside yarns and between yarns during VaRTM to the same degree as composites created from original fabrics. At the same time, initial thickness of nine-ply FGRPs is equivalent to that of six-ply composites made from recycled fiberglass. SEM images show that thickness of yarns in recycled composite is 100–150 μm larger and fibers packing inside yarn is less dense compared to composite with virgin fiberglass. The matrix areas between elementary fibers are 25–35 µm in size (Figure 6e). ImageJ (1.54g) software is used to calculate the volume ratio of the elementary fibers in the yarn. The initial FGRP had a 64% volume ratio, while the FGRP obtained from the fillers recovered after boiling in pyridine had a lower volume ratio of 51%. These findings were supported by TG analysis results, which showed that the amount of matrix in composites made with recycled filler has increased by 23%.

In Figure 6b, two distinct phases were identified. Phase 1 corresponded to the portion of the matrix obtained after repetitive VaRTM. Phase 2 corresponded to the residual matrix adjacent to the fibers, representing unremoved original polymer. Microhardness testing of the isolated phases revealed that phase 2 had a hardness 1.8 times greater than phase 1 (Figure S4).

The next step was to test the flexural strength of the composites. Results presented at Table 3. Due to the abovementioned phenomena, a 29.7% decrease in the bending strength characteristics was observed for samples based on recycled material.

**Figure 7:**
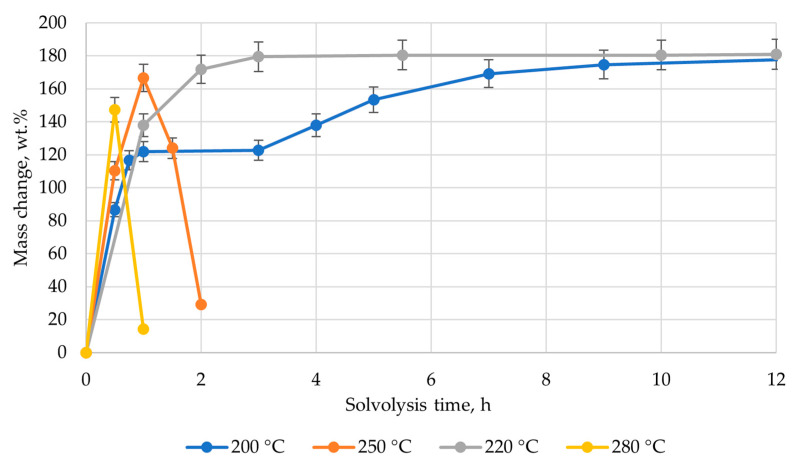
Plot of composite material mass change during solvolysis process under subcritical pyridine over time at the different temperatures.

In order to improve the quality of regenerated filler, a study was conducted on solvolysis of epoxy/fiberglass in pyridine via an autoclave. Composite samples (2 g) were used for this purpose. The process was carried out with subcritical pyridine at temperatures ranging from 200 to 300 °C. Due to the high energy consumption, further increases in temperature were not considered. The critical temperature of pyridine corresponds to a value of 346.8 °C at a pressure of 5.63 MPa. A plot of the mass change in composite samples over time during the solvolysis process was obtained at different temperatures, based on experimental data (Figure 7).

**Figure 8:**
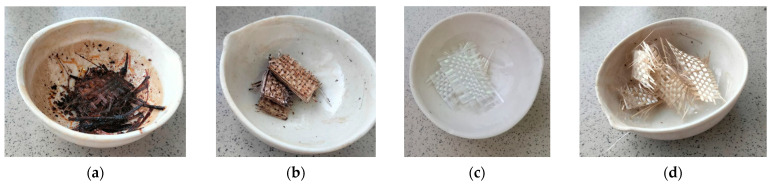
Photos of recovered fiberglass samples under different temperatures in autoclave: (**a**) 200 °C, 15 h; (**b**) 220 °C, 12 h; (**c**) 250 °C, 2 h; (**d**) 280 °C, 1 h.

In all cases, the recovered reinforcing fillers were obtained at different temperatures (Figure 8). In the case of processing at temperatures of 200 and 220 °C, polymer particles remained on the surface of fibers, similar to the case with treatment at the boiling point under atmospheric pressure. The resulting curves were identical to the swelling curves. After two hours of treatment at 220 °C, the mass change remained constant. Also, the graph of the mass change in the composite sample during solvolysis at 200 °C was characterized by the presence of a step, after which the parameter remained constant after reaching 7 h.

**Figure 9:**
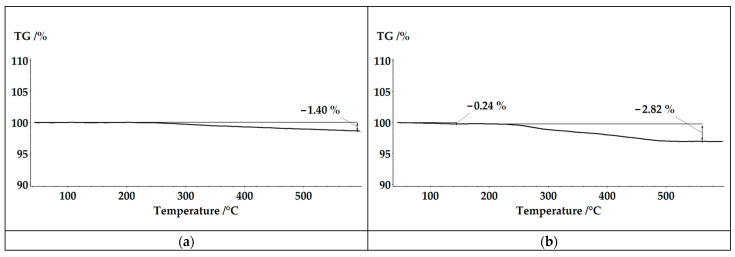
TG analysis of fiberglass recovered: (**a**) at 250 °C, 2 h; (**b**) at 280 °C, 1 h.

**Figure 10:**
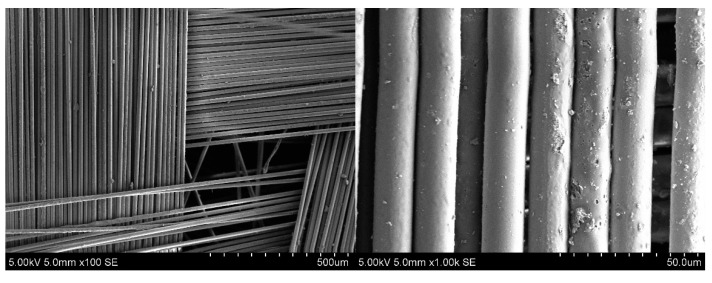
SEM of fiberglass recovered at 250 °C, 2 h.

During the process, at a temperature of 250 °C, after 2 h, sheets of purified white fiberglass were obtained. The pressure in the reactor reached up 2.5 MPa. The plot of mass change showed the maximum at one hour after the start of the process. According to TG analysis, the residual content of degradation products on fibers was 1.4 wt.% (Figure 9). The study of the fiber structure showed that its average diameter is 14.8 µm, which is larger than the initial fiber by 0.4 µm. Fiber surfaces exhibited pores ranging from 1.6 µm to 0.2 µm in size (Figure 10). Based on the data and results presented earlier [44], it is possible to assume that a polymer coating was formed on the surface of fibers.

Solvolysis was also carried out at a temperature of 280 °C. In this case, the duration of the process was 1 h. The maximum on the graph of mass change is recorded after 0.5 h (Figure 7). The residual content of degradation products on the fiber surface according to TG data was 2.82 wt.% (Figure 9). Also, at this figure, a small step was observed at a temperature of 140 °C, which corresponds to the evaporation of pyridine. The greater amount of organic products on the fiber surface is due to the washing conditions.

The results of the tensile strength of the fibers are presented in Table 2. It was discovered that the tensile strength of individual glass fibers recovered in pyridine solution decreased slightly. These results confirmed that elevated temperatures negatively effected the strength characteristics of glass fibers, as previously reported [47].

Fibers recovered at 280 °C exhibited a residual tensile strength of 91%. While the strength of the fibers recovered in pyridine solution for two hours at 250 °C was 2715 MPa, which is 7% lower than the original strength. The glass fiber samples recovered at temperatures of 200 and 220 °C exhibited substantial amounts of polymer matrix residues. These fabrics cannot be used for product manufacturing despite their strength characteristics. This fact is confirmed by the results of studies of composites obtained at the boiling point of pyridine. Thus, the best conditions for recovering fibers were achieved through the process of solvolysis at 250 °C for two hours.

**Figure 11:**
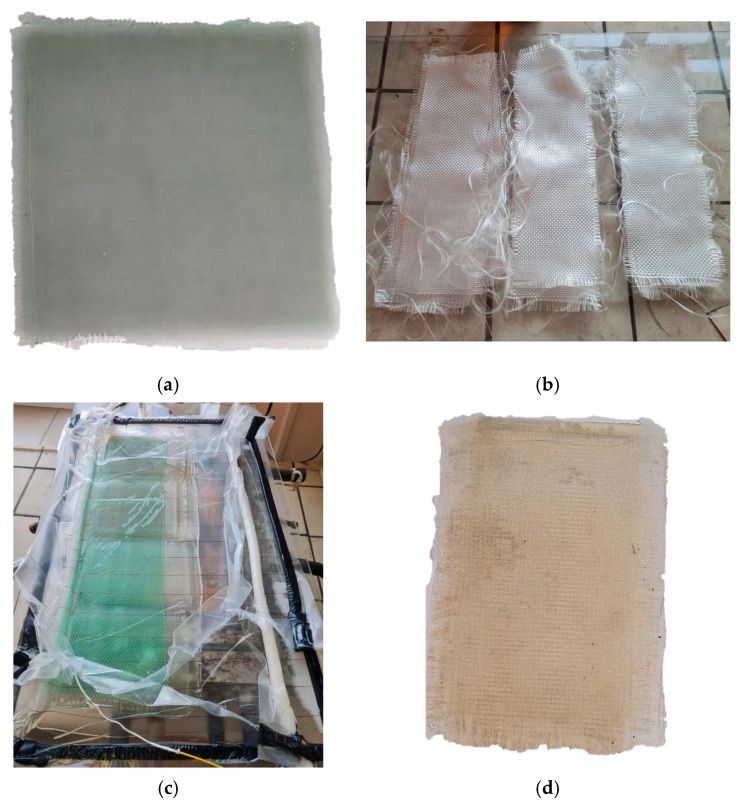
Recycling of FGRP at 250 °C, 2 h: (**a**) original FGRP; (**b**) recovered fiberglass fabric, (**c**) VaRTM of recovered fiberglass; (**d**) recovered FGRP.

**Figure 12:**
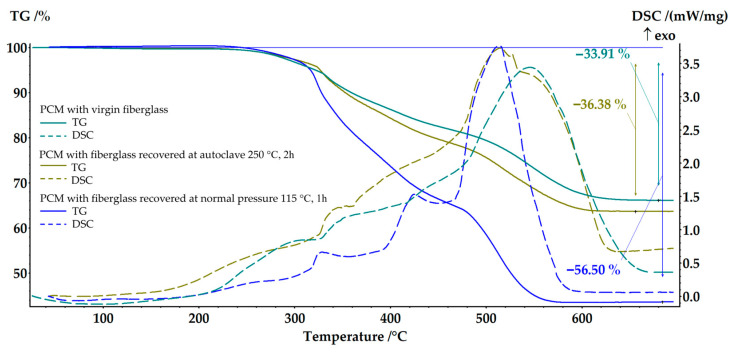
TG-DSC analysis of PCM with different fillers.

Photographs of recovered 10 × 30 cm fiberglass samples demonstrated in Figure 11b. The fabrics were cut to a size of 10 × 15 cm. The recovered fabrics were used to obtain samples of polymer composite materials based on the epoxy matrix (Figure 11). It was found that the amount of the matrix in that plastic was 36.38 wt.% via TG analysis. This result was almost like the sample based on virgin fibers (33.91 wt.%) (Figure 12). The structural analysis (Figure 6c,f) also showed a uniform distribution of fibers in the composite material. The volume ratio of elementary fibers in the fiber was 60% (Figure 6f). In contrast, the original composite contained matrix fragments between the elementary fibers of the fiber, as shown in Figure 6b. However, these fragments measured only 5–26 µm.

Composites made from recovered fillers showed increased flexural strength. The flexural strength increased by 16.1% for composites made from fabric that was recovered in pyridine for two hours at 250 °C. A study was conducted to investigate the effect of acetone, which was used to wash regenerated fabrics, and solvolysis liquid to understand the cause of this significant strengthening in polymer composites due to the use of recovered reinforcements. For this purpose, virgin fiberglass was immersed in various solvents. The treatment involved placing fiberglass samples in a vessel with a solvent and shaking intensively for 1 min. The treated fabrics were then dried to remove volatile components at a temperature of 120 °C for two hours. After that, they were used to produce FGRP through the VaRTM process. The composite sample produced from fiberglass, which was washed in acetone, has a strength that is 18.8% greater than the reference sample. Igleseas et al. [63] presented the study on the effects of hydrolytic damage during aging of fiberglass sizing. The use of a solvent made it possible to effectively remove such impurities, improving the contact between the fiber and the resin.

The composite, made of reinforcing filler, which was briefly immersed in washing acetone with a small amount of polymer matrix degradation products and pyridine, exhibited a strength 32.7% greater than that of the reference sample. Additionally, a similar result was achieved for a composite sample made from fabric treated with a solvolysis liquid and then washed with acetone. The strength of the sample increased by 28.7%. This effect can be achieved by modifying the adhesive material with solvolysis products [64] and pyridine [63]. This may be caused by the formation of additional bonds between the sizing and the matrix, as well as the polymerization of pyridine [65]. Thus, the resulting strengthening effect in composite materials made from regenerated fiberglass was attributed to residual degradation products from the epoxy matrix on the fiber surface. The flexural strength of composites with regenerated fiberglass fabrics was 90.2% of that of a sample based on virgin fiberglass treated with a solvolysis liquid and then washed with acetone. This value correlates well with the residual strength of fibers obtained through solvolysis treatment.

## 4. Conclusions

In this study, the solubility parameter was used to support the choice of solvent for the solvolysis process. It was demonstrated that nitrogen-containing solvents were highly effective for the solvolysis of epoxy systems. The swelling process of an epoxy polymer based on bisphenol-A and -F, which were cured with a multifunctional amine hardener, was investigated at room temperature. Based on the results of the experiment, pyridine was found to be the best choice for use as a solvent in the solvolysis process. The epoxy matrix was effectively swollen and degraded by pyridine. Maximum swelling of 38% was achieved at room temperature after 140 h. Increasing solvolysis temperature accelerated destructive processes. Solvolysis at its boiling point (115.2 °C) for one hour yielded recovered fiberglass. The recovered fibers retained a strength of 91% of the original fibers’ strength. FGRP using this recovered fiberglass showed a lower flexural strength of 295.4 MPa.

Autoclave-assisted solvolysis in pyridine under subcritical conditions was realized. Recovered fiberglass fabric sheets were produced at temperature of 250.0 °C. The residual polymer content was 2.8%. The residual tension strength of the recovered fibers was 93%. These fabrics were used to produce composites. The flexural strength of FGRPs increased by 16.1% (to 539.0 MPa) compared to composites made with virgin fabrics.

Also, the influence of solvolysis and washing liquids was investigated. Acetone treatment increased flexural strength of FGRP by 18.8%, obtained from virgin fabrics. Composites made from fiberglass treated with solvolysis liquid exhibited a 32.7% increase in flexural strength.

Thus, the results obtained from this study demonstrate the potential of using the solvolysis method to recycle thermosetting polymer composites. This method allows us to obtain recycled reinforcing filler with high residual strength. This makes it possible to create composite materials with properties similar to those based on virgin reinforcing fillers. The current study has promising results, but further research is needed to fully understand the environmental impact of using pyridine in the solvolysis process. In addition, it would be useful to continue research in order to find an effective solvent for a lower-temperature solvolysis process.

## Figures and Tables

**Table 1:** The results of the calculation of the solubility parameter.

Solvent	Δ, (cal/cm^3^)^0.5^	Δ*δ*, (cal/cm^3^)^0.5^
Fragment of epoxy polymer	10.095	-
Aniline	10.95	0.855
Benzene	9.3	−0.795
Xylene	8.7	−1.395
Toluene	8.9	−1.195
Pyridine	10.1	0.005
i-Butanol	10.9	0.805
Butyric acid	11.2	1.105
n-Butanol	11.3	1.205
Methanol	14.8	4.705
Ethanol	13.0	2.905
Acetic acid	12.8	2.705
Phenol	12.1	2.005

**Table 2:** Properties of recovered glass fibers.

No.	T, °C	Time, h	Glass Fiber Tensile Strength, MPa	TG, wt.%
1	200	10	-	18.5
2	220	5	-	11.9
3	250	2	2715	1.4
4	280	1	2645	2.8
0	-		2918	2.1
	boiling	1	2654	20.4

**Table 3:** FGRP flexural strength.

Filler	Treatment	FGRP Flexural Strength, MPa
Origin fabric	-	413
	Acetone treatment	551.6
	Acetone after wash of recovered fabrics	616.4
	Solvolysis liquid and acetone wash	597.6
Recovered	Pyridine, 1 h at the boiling point	295.4
	2 h 250 °C	539

## Data Availability

All related additional data are available in the Supplementary Materials.

## Supplementary Materials

The following supporting information can be downloaded at: https://www.mdpi.com/article/10.3390/polym17111513/s1, Figure S1: FTIR of the initial epoxy matrix; Figure S2: FTIR of residues on the fiberglass surface; Figure S3: FTIR of the sediment; Figure S4: SEM of HMV indentation imprints for phases 1 and 2.
