# Supplementary figures and images for: Recycling of Epoxy/Fiberglass Composite Using Pyridine
Source: Polymers (Basel). 2025 May 29;17(11):1513. doi: 10.3390/polym17111513 (PMC12157202; doi:10.3390/polym17111513)

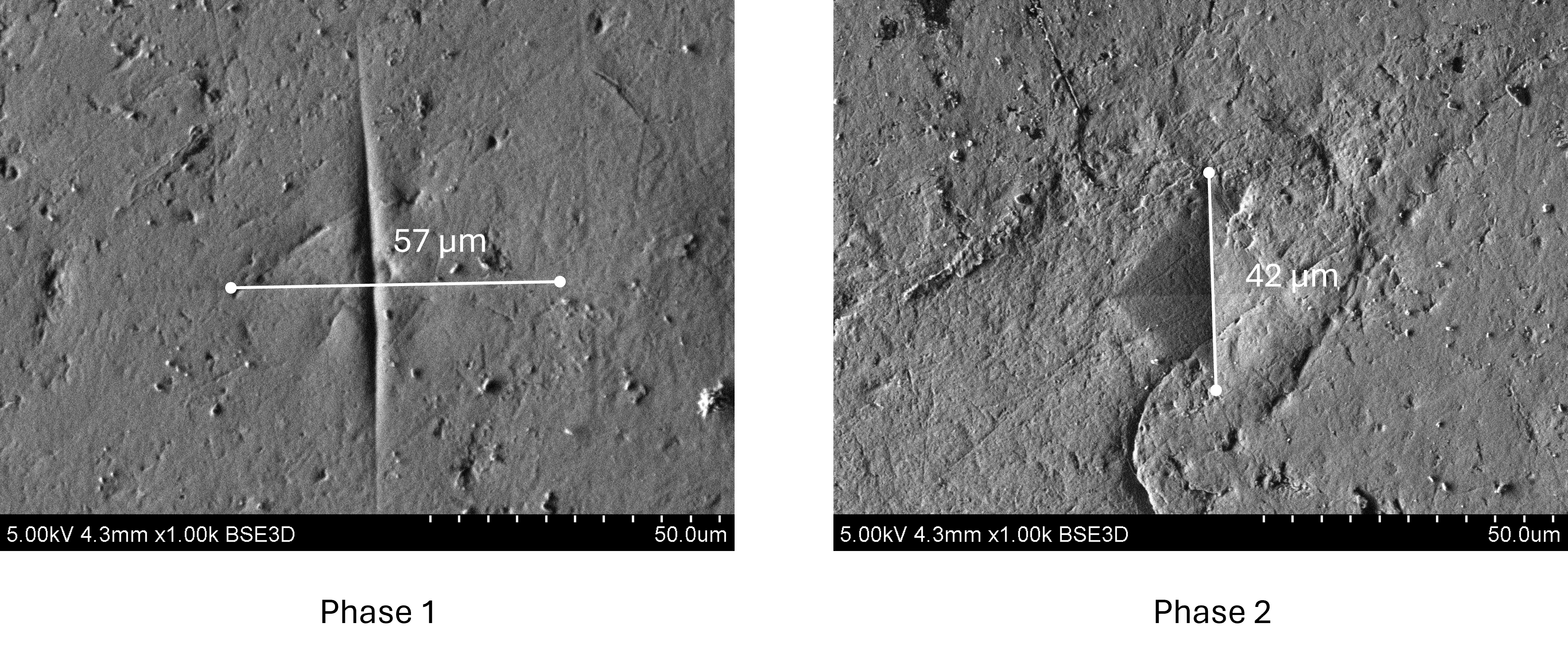

Supplement: Supplementary file 1 [file polymers-17-01513-s001.zip › Figure S4.tif]

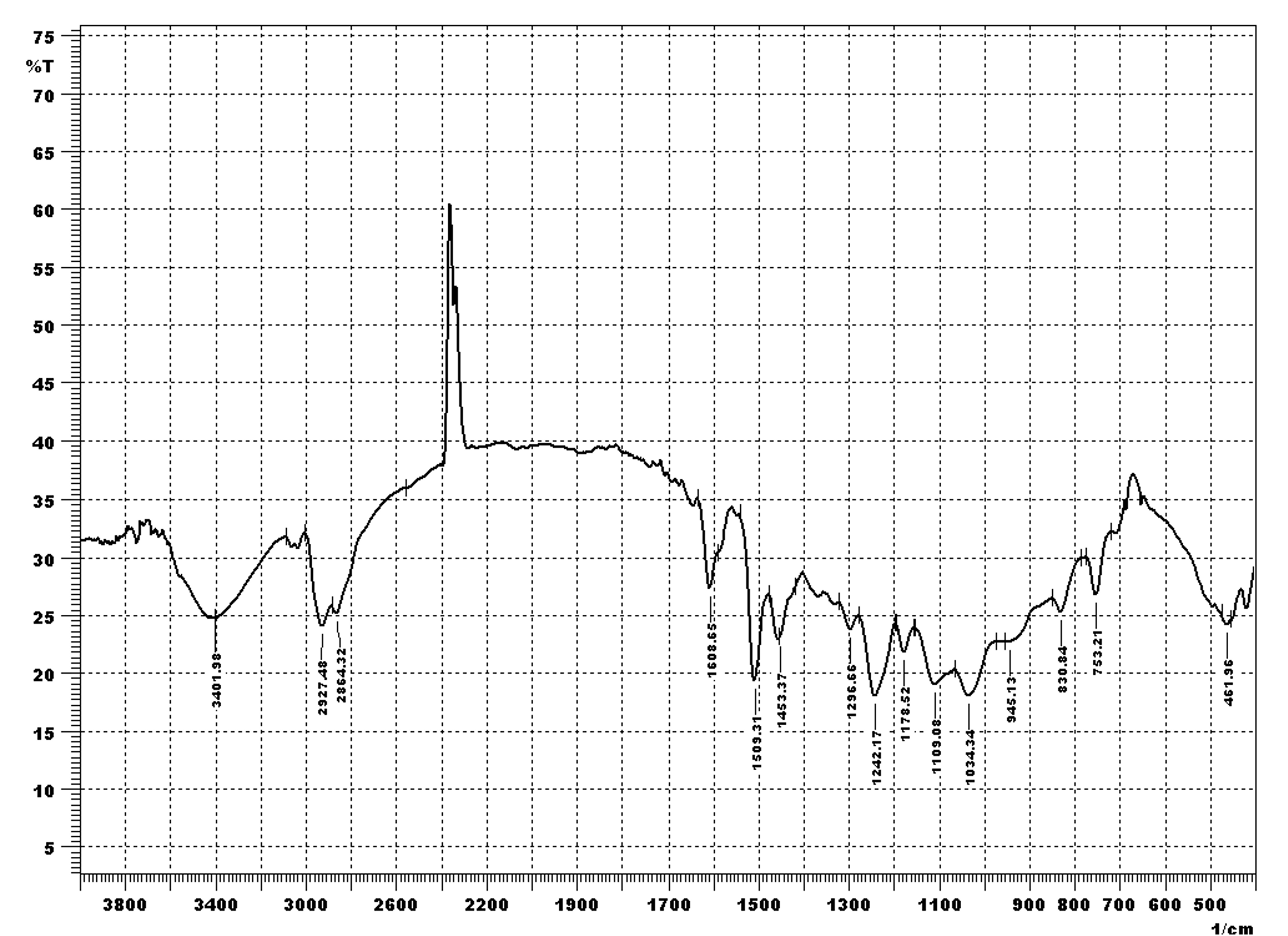

Supplement: Supplementary file 1 [file polymers-17-01513-s001.zip › Figure S1.tif]

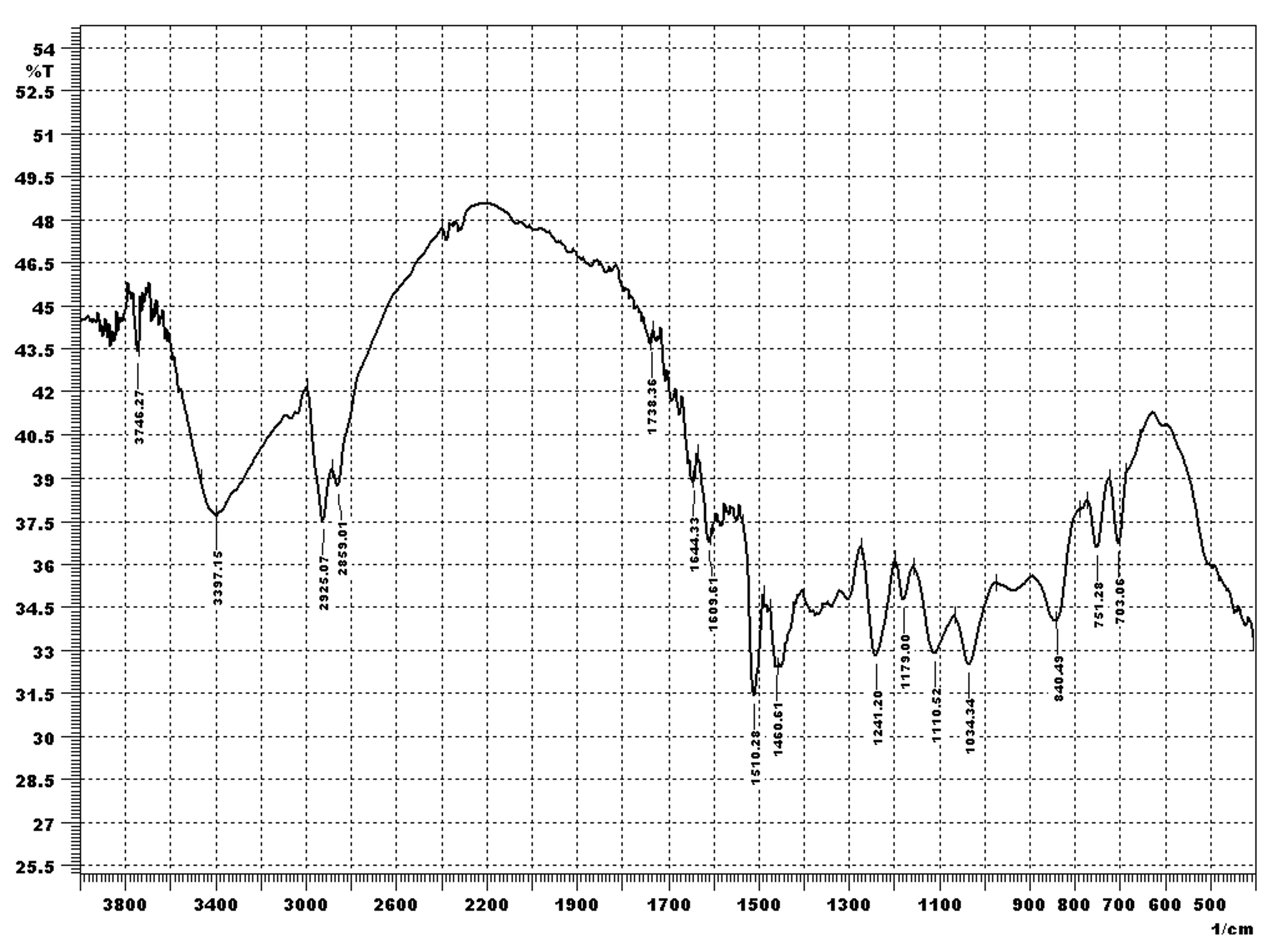

Supplement: Supplementary file 1 [file polymers-17-01513-s001.zip › Figure S2.tif]

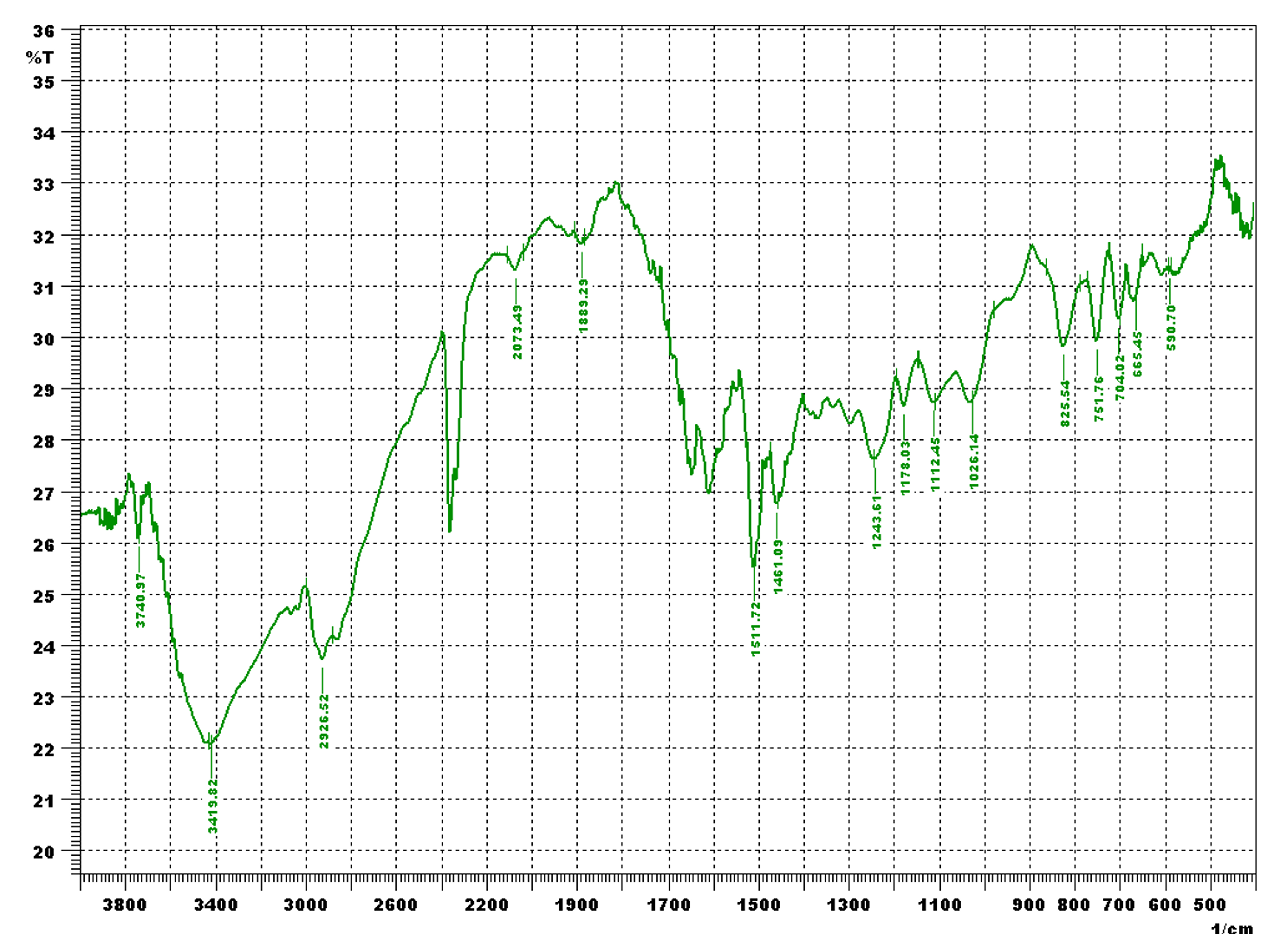

Supplement: Supplementary file 1 [file polymers-17-01513-s001.zip › Figure S3.tif]
